# Long-Term Varicella Zoster Virus Immunity in Paediatric Liver Transplant Patients Can Be Achieved by Booster Vaccinations—A Single-Centre, Retrospective, Observational Analysis

**DOI:** 10.3390/children9020130

**Published:** 2022-01-19

**Authors:** Tobias Laue, Elisabeth Oms, Johanna Ohlendorf, Ulrich Baumann

**Affiliations:** Division of Paediatric Gastroenterology and Hepatology, Department of Paediatric Liver, Kidney and Metabolic Diseases, Hannover Medical School, 30625 Hannover, Germany; Elisabeth.Oms@helios-gesundheit.de (E.O.); Ohlendorf.Johanna@mh-hannover.de (J.O.); Baumann.U@mh-hannover.de (U.B.)

**Keywords:** paediatric liver transplantation, chronic liver disease, vaccination, immunization, varicella, VZV, immunosuppression, chickenpox

## Abstract

Varicella is one of the most common vaccine-preventable infections after paediatric solid organ transplantation; thus, vaccination offers simple and cheap protection. However, children with liver disease often progress to liver transplantation (LT) before they reach the recommended vaccination age. As a live vaccine, varicella zoster virus (VZV) vaccination after transplantation is controversial; however, many case series demonstrate that vaccination may be safe and effective in paediatric liver transplant recipients. Only limited data exists describing long-term vaccination response in such immunocompromised patients. We investigated retrospectively vaccination response in paediatric patients before and after transplantation and describe long-term immunity over ten years, including the influence of booster-vaccinations. In this retrospective, single-centre study, 458 LT recipients were analysed between September 2004 and June 2021. Of these, 53 were re-transplantations. Patients with no available vaccination records and with a history of post-transplant lymphoproliferative disease, after hematopoietic stem cell transplantation and clinical chickenpox were excluded from this analysis (*n* = 198). In total, data on 207 children with a median annual follow-up of 6.2 years was available: 95 patients (45.9%) were unvaccinated prior to LT. Compared to healthy children, the response to vaccination, measured by seroconversion, is weaker in children with liver disease: almost 70% after one vaccination and 93% after two vaccinations. One year after transplantation, the mean titres and the number of children with protective antibody levels (VZV IgG ≥ 50 IU/L) decreased from 77.5% to 41.3%. Neither diagnosis, gender, nor age were predictors of vaccination response. Booster-vaccination was recommended for children after seroreversion using annual titre measurements and led to a significant increase in mean titre and number of protected children. Response to vaccination shows no difference from monotherapy with a calcineurin inhibitor to intensified immunosuppression by adding prednisolone or mycophenolate mofetil. Children with liver disease show weaker seroconversion rates to VZV vaccination compared to healthy children. Therefore, VZV-naïve children should receive basic immunization with two vaccine doses as well as those vaccinated only once before transplantation. An average of 2–3 vaccine doses are required in order to achieve a long-term seroconversion and protective antibody levels in 95% of children.

## 1. Introduction

Paediatric liver transplantation is a well-established procedure in patients with chronic as well as acute liver failure. Five-year survival rates are over 90% [1,2]. However, there is a need for lifelong immunosuppression and a risk of infection [1,2,3]. Chickenpox ranks third as a vaccine-preventable infection (VPI) after paediatric solid organ transplantation [4].

An initial infection with varicella zoster virus (VZV) is often mild in immunocompetent children, with a fever and skin rash, but severe cases involving the central nervous system or pneumonia have been reported [5]. By contrast, case reports also describe severe cases with visceral involvement (e.g., pneumonitis, hepatitis, or meningoencephalitis) and even death in immunocompromised patients [6]. Case series in liver-transplanted children mainly describe skin involvement where treatment with varicella-zoster immunoglobulin (VZIG) and/or acyclovir was begun [7,8]. However, prolonged hospital stays with multiple organ failure [9] as well as cases of death related to VZV infection in liver transplant recipients have also been reported [10].

Vaccination offers simple and cheap protection. In the USA, VZV vaccination is recommended from 12 months [11] and in Germany from 11 months [12], but if an urgent transplant is necessary, it can be administered from 6 months of age [13]. Compared to paediatric kidney [14] and lung [15] transplantation, liver transplant patients are on average younger. Between 20 and 30% are transplanted in their first year of life [2,16]. As a result, the window of opportunity for live vaccinations is usually limited, and children often do not reach the recommended minimum age. Around 88% of U.S. paediatric patients were up-to-date with VZV vaccination at time of transplant [17], and under two-thirds of children with chronic liver disease were vaccinated age-appropriately in observation of the European Reference Network TransplantChild [18]. In adults, VZV immunity is demonstrated in over 96% of patients at time of liver transplantation—it remains open whether this is due to infection or vaccination [19]. In contrast, live vaccines are not generally recommended in patients after solid organ transplantation due to concerns that the immune system may fail to initiate a sufficient response [13], with the result that unvaccinated patients are subject to a high risk of infection in the long term and potentially leading to graft loss or death [20]. Recent studies suggest that immunization against varicella may be safe and effective even after paediatric liver transplantation [21,22]. However, there is few data on long-term VZV immunization in patients after paediatric liver transplantation.

This retrospective, observational, single-centre study analyses the immunization response to VZV in children and adolescents before and after liver transplantation. Antibody titres were examined depending on the number of VZV vaccinations prior to transplantation and the response to immunization in VZV-naïve children monitored. Furthermore, it should be determined how antibody levels behave over 10 years, including booster-vaccinations. Finally, it should be assessed whether intensified immunosuppression results in a poorer response and whether more vaccinations are required.

## 2. Materials and Methods

### 2.1. Patients and Data Acquisition

This single-centre, observational, retrospective study analysed children who underwent liver transplantation between September 2004 and June 2021 at Hannover Medical School (Germany). Only children with a certified immunization record and aged below 16 years at the time of transplantation were included in this study. If a child was re-transplanted more than 8 weeks after the first liver transplant, only data up to re-transplantation was used. Exclusion criteria were the need for hematopoietic stem cell transplantation, the development of a post-transplant lymphoproliferative disorder (PTLD), and/or the regular administration of immunoglobulins, and a history of chickenpox. The study was conducted according to the guidelines of the Declaration of Helsinki and approved by Hannover Medical School Ethics Committee (Statement N° 9928_BO_K_2021, approval date 6 August 2021).

Patients are usually seen every 6 months after the first year of paediatric liver transplantation. Standard immunosuppression includes ciclosporin A or tacrolimus. For intensified immunosuppression, this is supplemented with prednisolone or mycophenolate mofetil (MMF). Vaccination status is reviewed at annual check-ups. Any vaccinations carried out are added to the patient’s file. Varicella antibody titre is determined pre-transplant and annually after transplantation. Those measurements of patients who received albumin, fresh frozen plasma or immunoglobulins in the last 3 months before antibody measurement were excluded, as well as VZV titres of those children below 9 months of age due to potential maternal antibodies [23]. VZV-specific antibodies ≥ 50 IU/L were considered as immunity [24].

Vaccinations may be carried out again from the first check-up after paediatric liver transplantation. Recommendations are based on the number of previous VZV-vaccinations and titre: If no vaccination has yet been given, a basic immunization is recommended. If at least one dose has already been administered, a booster vaccination is recommended if the VZV titre is below 50 IU/L. After a biopsy-proven acute rejection with resultant steroid therapy, no vaccinations should be carried out for 3 months.

### 2.2. Statistical Analysis

Qualitative data is expressed as numbers and percentages (%). Quantitative data is expressed as median (25–75% quartile) or mean ± standard deviation (SD). The comparison of two groups with categorical variables was performed using chi-squared test or Fisher’s exact test. Mann–Whitney U test was used for continuous variables due to non-normality. Data was significant with *p*-values of *p* < 0.05 and marked as follows: * *p* < 0.05, ** *p* < 0.01, *** *p* < 0.001. Statistical analysis was performed using R version 4.0.5 [25]. For graphical data, ggplot2 package version 3.3.3 was used [26].

## 3. Results

### 3.1. Study Population

A total of 458 paediatric liver transplantations were performed during the analysis period from September 2004 to June 2021. Of these, 53 were re-transplantations, 183 patients could not be evaluated due to unclear vaccination data, and three children had a history of chickenpox without the need for inpatient treatment and total recovery. In total, vaccination records of 219 patients were available. Twelve were excluded from analysis: eight due to PTLD and four because of hematopoietic stem cell transplantation.

Of these 207 children 104 (50.2%) were female. A total of 46% of patients were diagnosed with biliary atresia (BA), followed by cryptogenic cirrhosis (10.6%), hepatic malignancy (10.1%), progressive familial intrahepatic cholestasis (PFIC) (8.2%), acute liver failure (8.2%), and metabolic conditions (6.3%). Median age at transplantation was 1.56 years (0.65–4.77). More than 54% were vaccinated against VZV at least once before liver transplantation, with a median age of 1.04 years, and 74 of 207 patients (35.7%) received a complete primary series of two doses. Patients were followed up for a median of 6.2 years. Baseline characteristics are presented in Table 1.

### 3.2. VZV Immunity in the First Year after Pediatric Liver Transplantion

**Figure 1:**
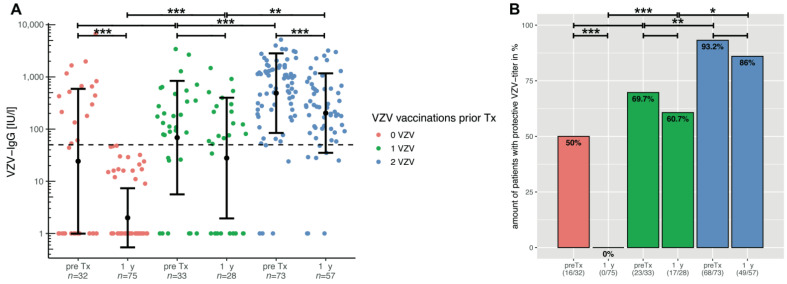
(**A**) VZV-specific antibodies before and 1 year after paediatric liver transplantation, depending on number of VZV vaccinations prior to transplantation. The *y*-axis is scaled logarithmically. Data is shown as mean ± standard deviation. The dashed line marks the threshold for serological protection. All groups show a decrease in mean titre after transplantation, regardless of the number of vaccine doses. However, as the bar plots in (**B**) show, patients with protective VZV antibodies after 1 vaccination are more than 60%, and after 2 vaccinations, more than 85% are still serologically protected. Significant data is marked as follows: * <0.05, ** <0.01, *** <0.001.

Analysis of VZV antibodies before transplantation show significantly higher mean titres after two doses of vaccination than after one or no vaccination (*p* < 0.00001 for both; Figure 1A) and sufficient titres in over 93% of children (Figure 1B). After one vaccination, only two-thirds of patients have a sufficient titre. In all groups, the mean titre drops after transplantation. Without vaccination, no patients have sufficient titres (*p* = 0.00001; Figure 1B). After one vaccination, almost 61% and after two vaccinations, 86% of all children and adolescents are still serologically protected (not significant; Figure 1B). Thus, more than 75% of all patients vaccinated at least once before transplantation still have protective titre levels afterwards.

### 3.3. VZV Immunization of VZV-Naïve Children after Transplantation and Titre

**Figure 2:**
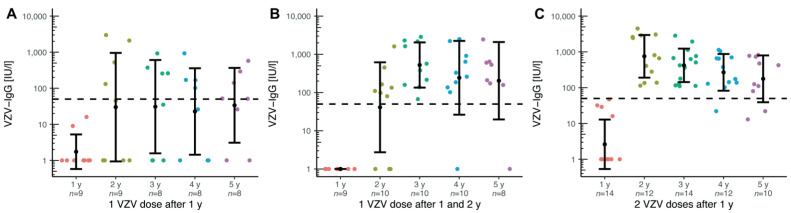
VZV-specific antibody responses following VZV-vaccination in VZV-naïve paediatric liver transplant recipients immunized for the first time 1 year after transplantation. Titre levels over 4 years, following (**A**) one dose after 1 year, (**B**) one dose after 1 and 2 years, and (**C**) two doses after 1 year. The *y*-axis is scaled logarithmically. Data is shown as mean ± standard deviation. The dashed line marks the threshold of serological protective titres.

As described above, almost 46% of our patients were not vaccinated against chickenpox before transplantation. In the next step, those unvaccinated children and adolescents who were immunized after the first annual check-up were examined. The titre is shown separately, depending on the number of vaccinations: Figure 2A shows the course after one vaccination. In Figure 2B, each patient received a vaccination dose after the first and second annual check-ups. For Figure 2C, data on two vaccinations was plotted after the first annual check-up. The vaccination response under immunosuppression is better after two vaccinations, even if the titre decreases over time.

### 3.4. Response to Booster Vaccinations in Pre-Transplant Vaccinated Patients without Protective Titres at the First Annual Check-Up

Of the 11 patients with one pre-transplant vaccination dose and without sufficient vaccine titres at the first annual follow-up, a total of six were boosted with a second vaccination dose. Of these, four (66.7%) showed seroconversion at the second annual check-up. The other two children only showed a serological response to the third vaccination. At the third annual check-up, protective VZV antibodies were detectable in all six children. Only eight of the 57 children (14%) with basic immunization with two vaccine doses had no protective VZV antibodies after transplantation. Of these, three (37.5%) received a VZV booster vaccination and had seroconversion at the second annual check-up.

### 3.5. Long-Term Observation of VZV-Specific Titres and Immunity over 10 Years with Vaccination Course

**Figure 3:**
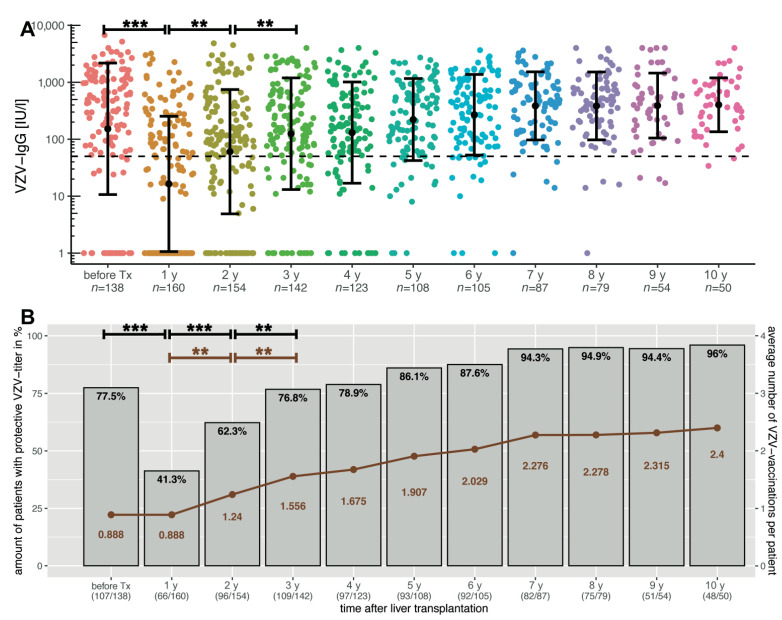
(**A**) VZV-specific antibody course in paediatric liver transplant recipients over ten years after transplantation. The *y*-axis is scaled logarithmically. Data is shown as mean ± standard deviation. The dashed line marks the threshold for serological protection. The titre dropped significantly up to the first annual check but increased over the next few years with the possibility of vaccination, even if this increase was only significant at the second and third annual check. This also affected the numbers of patients with protective VZV titres, as shown in the bar plots in (**B**). The brown graph shows the average number of VZV vaccinations per patient. This was stable from the seventh annual check-up on, when around 95% of all patients were serologically protected, which required an average of 2.3 vaccinations per patient. Significant data is marked as follows: ** <0.01, *** <0.001.

Figure 3A shows the VZV-titre course before transplantation and up to the tenth annual check-up. This shows a significant decrease from before transplantation to the first annual check-up (*p* < 0.00001). As vaccinations were allowed again from this point, the titre increased significantly from the first to the second (*p* = 0.00008) and from the second to the third measurements (*p* = 0.00854).

Regarding the number of patients (Figure 3B) who were serologically immune to chickenpox, there was also a significant decrease from before transplantation to the first annual follow-up (77.5% vs. 41.3%; *p* < 0.00001). With the possibility of VZV vaccination, the numbers of serologically protected children increased significantly up to the third annual check-up. Furthermore, the average number of VZV immunizations per patient increased significantly from the first to the second (*p* = 0.002) and from the second to the third annual check-up (*p* = 0.0083). However, it took seven years and an average of 2.3 immunizations per patient until almost 95% of patients were serologically protected.

### 3.6. Influence of Immunosuppression on VZV Titres

**Figure 4:**
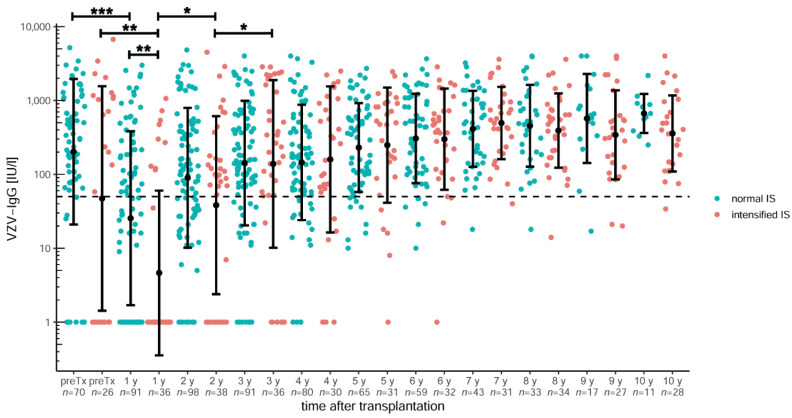
VZV-specific antibody course in paediatric liver transplant recipients over ten years after transplantation dependent on normal (monotherapy with ciclosporin A or tacrolimus) or intensified (monotherapy supplemented by prednisolone or mycophenolate mofetil) immunosuppression (IS). At the first annual follow-up after transplantation, the titres dropped significantly in both groups, and children with intensified immunosuppression also had a significantly lower titre compared with those with normal immunosuppression. At the second annual check-up, titres increased again and did not show any further difference. The *y*-axis is scaled logarithmically. Data is shown as mean ± standard deviation. The dashed line marks the threshold for serological protection. Significant data is marked as follows: * <0.05, ** <0.01, *** <0.001.

In order to investigate the influence of immunosuppression on the development of the VZV titres, immunosuppression was divided into normal (106 patients with a monotherapy with ciclosporin A or tacrolimus) and intensified (42 patients with monotherapy supplemented by prednisolone or mycophenolate mofetil), as shown in Figure 4. The classification on the basis of immunosuppression took place at the time of the second annual check-up, as from this point at the latest, immunosuppression remains stable and only rarely changes. Both groups did not differ in sex, number of VZV vaccinations prior to transplantation, and age at transplantation. In the normal immunosuppression group, 31 children (29.2%) received ciclosporin A, with a median trough level of 60 µg/L (IQR: 37–81). This was significantly higher at 70 µg/L (*n* = 20; IQR: 56–113) in the group with intensified immunosuppression (*p* = 0.03752), where 47% received ciclosporin. In contrast, the tacrolimus trough level showed no difference (*p* = 0.45326) between normal and intensified immunosuppression with 3.4 (IQR: 2.7–4.2) and 3.7 (IQR: 3.2–4.3), respectively.

The titres did not differ before transplantation (*p* = 0.25014). At the first annual check-up, they fell significantly in both groups (*p* < 0.00001 and *p* = 0.00932). Patients with intensified immunosuppression had significantly lower titres at the first annual check-up compared to normal immunosuppression (*p* = 0.0041) but showed a significant titre increase at the second and third annual check-ups (*p* = 0.03752 and *p* = 0.0455, respectively). However, comparing both groups over the remaining observation period of 10 years, there was no significant difference in the titre levels. Moreover, the average number of VZV vaccinations before and after liver transplantation, depending on immunosuppression, showed no significant difference between both groups.

## 4. Discussion

Varicella is a highly contagious virus and one of the most common vaccine-preventable infections following paediatric solid organ transplantation [5]. Although vaccination is an easy and cheap form of prevention, immunization status at transplantation is often not appropriate [17,18], so post-transplant immunizations would appear beneficial for this high-risk group. To the best of our knowledge, this is one of the largest retrospective, long-term studies on vaccination and antibody development in relation to varicella in paediatric liver transplant recipients.

As previously shown in other studies, the serological response to VZV vaccination before transplantation in children with liver disease is reduced; in a study by Donati et al. in 2000, only three of 11 children developed antibodies after one dose of varicella vaccine before transplantation [27]. A recently published study showed seroconversion in eight of 12 children to one vaccination and in 70% to two vaccinations, respectively [28]. Our data shows that more than 75% of all patients VZV vaccinated at least once before transplantation were serologically protected at the time of transplantation. This is in line with a study from Wu et al., where only 74% of all children with biliary atresia showed seropositive antibodies after vaccination [29]. Almost half of all patients included in our study had biliary atresia, which other recent studies described as an immunological dysregulation of B cells and the cause of the disease [30]. By contrast, healthy children showed a serological response of 85% to one vaccination and above 99% to two vaccinations [31]. In addition, a study in 555 adults showed serological immunity in over 96% at time of liver transplantation [19]. This underlines the poor serological response to VZV vaccination in children with liver disease.

Only just over 40% of all our patients still have protective VZV titres after liver transplantation (Figure 3B). This significant decrease may be due to the initially higher immunosuppression in the first year as well as antibody loss during and after surgery. One limiting factor is that around 50% of the unvaccinated VZV population also has sufficient titres before transplantation. Although measurements from patients who had previously received fresh frozen plasma or immunoglobulins were excluded as well as those patients with a history of chickenpox. Thus, this leaves open how many patients certainly had serological protective titres before transplantation and how strong the decline really is. Interestingly, Yoeli et al. showed that VZV non-immune transplant patients were younger at transplantation, received fewer doses, and had less time between vaccination and transplantation [32]. By contrast, healthy children vaccinated at an early age also had an increased risk of vaccine failure [33].

However, children with chronic liver disease often do not reach the age to start live vaccinations or are in such a poor general condition that vaccination can no longer be carried out. As a result, almost 46% of all of our patients examined were not vaccinated against chickenpox before transplantation. Although there is no general vaccination recommendation for live vaccines after solid organ transplantation [13], recent studies show that vaccination against chickenpox in paediatric liver transplant recipients is safe and immunogenic [21,22]. Our centre recommends a basic immunization for VZV-naïve children after the first annual check-up. As shown in Figure 2, not only is the mean titre higher after two vaccinations, but there are also serological non-responders after only one VZV vaccination. Even in healthy children, varicella cases have been reported after just one vaccination, possibly due to primary vaccine failure [34]. Moreover, they show a better response to two vaccinations [31]. In contrast, a study in which adult varicella-seronegative transplant recipients were immunized with recombinant herpes zoster, only 55% of patients showed a seroresponse [35]. Thus, basic immunization with two doses of vaccine is recommended in paediatric liver transplant recipients.

Nevertheless, the mean titre decreases over time (Figure 2). This is in line with observations by Verolet et al., who described a decline of VZV titre in the long-term in children following liver transplant. They identified a VZV IgG below 400 UI/L after one or two vaccine doses as a risk factor and a need for a supplementary vaccination dose [36]. We usually recommend a booster vaccination for patients with a titre below 50 IU/mL at annual check-up. As a result, mean titre, the number of protected children, and the average number of vaccinations per patient increased significantly for the second and third annual check-ups (Figure 3A). However, it takes seven years and an average of 2.3 vaccinations per person until almost 95% of patients have sufficient VZV titres (Figure 3B). This remains stable from the seventh to the tenth check-up. Verolet et al. demonstrated protection in 96% of patients after at least two VZV doses, with a median follow-up of 5.5 years [36]. As a consequence, basic immunization should be given to VZV-naïve children. Even after receiving one vaccination dose before transplantation, two vaccination doses after the first annual control seem to be recommended for long-term antibody maintenance and protection. If basic immunization could be completed before transplantation, a booster vaccination should be given depending on the antibody level.

Mycophenolate mofetil (MMF) inhibits B- as well as T-cell function [37] and is often used for intensified immunosuppression (e.g., after rejection) [38]. Interestingly, MMF therapy appears to reduce the response to hepatitis B immunization in children with rheumatoid arthritis [39]. Contrary to expectations, an intensified immunosuppression by adding prednisolone or MMF to a calcineurin inhibitor only seems to reduce the mean titre within the first year after liver transplantation (Figure 4). In the long term, this equalizes again. There is also no significant difference in the average number of vaccinations per patient.

In addition to the fact that this is a retrospective study, there are numerous limitations: With the sole measurement of antibodies, cellular immunity, which is also addressed by vaccination, is not taken into account [40]. The response to co-vaccination (two vaccines administered concurrently) and combination vaccines (measles, mumps, rubella, including VZV) before transplantation was not examined here but should have a similar response in the healthy population [41]. In addition, there are various approved VZV vaccines in Germany; however, these should be equally effective [42]. Additionally, in Germany, apart from mandatory measles vaccination for nursery and school-age children (since March 2020 [43]), all other vaccinations are only recommended. It remains unclear how many parents do not want to implement this and whether general practitioners follow them up, especially as immunization practices among paediatric transplant hepatologists already differ [44].

In conclusion, both before and after paediatric liver transplantation, two VZV vaccine doses are recommended for VZV-naïve children. If the interval before LT is only sufficient for one vaccination, this offers more than 60% serological protection up to a year later, but also underlines the poorer response in the context of liver disease compared to healthy children. After transplantation, annual serological measurement offers the opportunity for recommended vaccination updates. However, a booster vaccination is often required in these children in order to achieve sufficient, long-term seroconversion in almost 95% of patients. Therefore, for patients vaccinated once before transplantation, another two vaccine doses after transplantation are advisable. Those with completed basic immunization, one booster vaccination after transplantation seems to be recommendable according to VZV-titre. In children with intensified immunosuppression, the positive effect of such booster vaccinations is maintained.

## Figures and Tables

**Table 1:** Patient data of 207 children with an available vaccination record who underwent liver transplantation between September 2004 and June 2021 at Hannover Medical School (Germany).

	All Children (*n* = 207)
Gender, female (%)	104 (50.2%)
Diagnosis	Biliary atresia: 96 (46.4%) Cryptogenic cirrhosis: 22 (10.6%) Hepatic malignancy: 21 (10.1%) Acute liver failure: 17 (8.2%) PFIC: 17 (8.2%) Cystic fibrosis: 8 (3.9%) Alagille syndrome: 7 (3.4%) Citrullinemia: 4 (1.9%) Neonatal hemochromatosis: 3 (1.4%) DGUOK deficiency: 2 (1.0%) Methylmalonic aciduria: 2 (1.0%) Alpha-1 antitrypsin deficiency: 2 (1.0%) Argininosuccinate lyase deficiency: 1 (0.5%) Ornithine transcarbamylase deficiency: 1 (0.5%) Glycogen storage disease type IV: 1 (0.5%) Congenital bile acid synthesis defect: 1 (0.5%) Amanita phalloides poisoning: 1 (0.5%)
Year of birth, median (IQR)	2011 (2007–2015)
Number of VZV doses prior to transplantation	0 vaccinations: 95 (45.9%) 1 vaccination: 38 (18.4%) 2 vaccinations: 74 (35.7%)
Age at 1st VZV vaccination prior to transplantation, median (IQR)	1.04 (0.94–1.39)
Age at 2nd VZV vaccination prior to transplantation, median (IQR)	1.49 (1.23–2.57)
Age at time of transplant, median (IQR)	1.56 (0.65–4.77)
Year of liver transplantation, median (IQR)	2014 (2010–2018)
Follow-up in years, median (IQR)	6.2 (2.0–9.5)
Age at 1st VZV vaccination after transplantation, median (IQR)	3.22 (2.18–5.62)
Age at 1st VZV vaccination after transplantation for VZV-naïve children, median (IQR)	2.70 (2.00–3.51)

## Data Availability

All data requests should be submitted to the corresponding author for consideration. Access to anonymized data may be granted, following review.
